# Favorable prognostic role of tropomodulins in neuroblastoma

**DOI:** 10.18632/oncotarget.25491

**Published:** 2018-06-05

**Authors:** Paola Bettinsoli, Giulia Ferrari-Toninelli, Sara Anna Bonini, Michela Guarienti, Davide Cangelosi, Luigi Varesio, Maurizio Memo

**Affiliations:** ^1^ Department of Molecular and Translational Medicine, University of Brescia Medical School, Brescia, Italy; ^2^ Laboratory of Molecular Biology, Giannina Gaslini Institute, Genova, Italy

**Keywords:** neuroblastoma, tropomodulins, favorable prognostic biomarkers, overall survival probability, therapeutic strategies

## Abstract

Neuroblastoma is a pediatric tumor of the sympatoadrenal lineage of the neural crest characterized by high molecular and clinical heterogeneity, which are the main causes of the poor response to standard multimodal therapy. The identification of new and selective biomarkers is important to improve our knowledge on the mechanisms of neuroblastoma progression and to find the targets for innovative cancer therapies. This study identifies a positive correlation among tropomodulins (TMODs) proteins expression and neuroblastoma progression. TMODs bind the pointed end of actin filaments, regulate polymerization and depolymerization processes modifying actin cytoskeletal dynamic and influencing neuronal development processes. Expression levels of TMODs genes were analyzed in 17 datasets comprising different types of tumors, including neuroblastoma, and it was demonstrated that high levels of tropomodulin1 (*TMOD1*) and tropomodulin 2 (*TMOD2*) correlate positively with high survival probability and with favorable clinical and molecular characteristics. Functional studies on neuroblastoma cell lines, showed that TMOD1 knockin induced cell cycle arrest, cell proliferation arrest and a mature functional differentiation. TMOD1 overexpression was responsible for particular cell morphology and biochemical changes which directed cells towards a neuronal favorable differentiation profile. TMOD1 downregulation also induced cell proliferation arrest but caused the loss of mature cell differentiation and promoted the development of neuroendocrine cellular characteristics, delineating an aggressive and unfavorable tumor behavior. Overall, these data indicated that TMODs are favorable prognostic biomarkers in neuroblastoma and we believe that they could contribute to unravel a new pathophysiological mechanism of neuroblastoma resistance contributing to the design of personalized therapeutics opportunities.

## INTRODUCTION

Neuroblastoma is an embryonic tumor of the peripheral sympathetic nervous system, arising during fetal or early postnatal life from sympathetic cells derived from the neural crest. It is the most common solid extra cranial malignancy of childhood affecting 10.5 per million children between 0 and 14 years of age and it is responsible for 15% of all cancer-related mortalities in children [1, 2]. Neuroblastoma is characterized by a broad range of clinical and biological heterogeneity, ranging from spontaneous regression or differentiation into benign tumors to rapid and aggressive phenotype that does not respond to current intensive multimodal therapy. In half of cases distant metastasis are detected at diagnosis and the tumor is classified as “high-risk” with a survival rate lower than 40% [3]. Several gene mutations, deletions or chromosomal rearrangements are involved in the onset of neuroblastoma and are associated with poor outcome, such as *ALK*, *PHOX2B* and *MYCN* gene amplification [4, 5]. Our previous study identified Notch pathway as one of the molecular pathways involved in the onset and progression of neuroblastoma [6]. In particular, we recognized Notch ligand DLL1 as the specific molecular target in childhood neuroblastoma and we proposed miRNAs as novel therapeutic tool to attack “DLL1 positive” neuroblastoma [7]. The challenge is to identify innovative and selective biomarkers to better understand clinical and molecular mechanisms underlying neuroblastoma progression and to devise new personalized therapeutics opportunities. In this study we demonstrated a strong correlation between TMODs expression and neuroblastoma survival. TMODs are a conserved family of 40 kDa proteins that cap actin filaments pointed end, stabilize filaments and inhibit their disassembly and turnover; they are defined *pointed end capping protein for actin filaments* [8, 9]. There are four TMOD isoforms (TMOD1, TMOD2, TMOD3, TMOD4) expressed in different tissues in vertebrates encoded by distinct genes. TMOD1 and TMOD2 isoforms are the only expressed in neurons, TMOD3 is ubiquitous and TMOD4 is mainly localized in skeletal muscle cells [10]. TMODs interact with a series of cytoskeletal proteins and this link allows to modulate the polymerization and depolymerization of actin monomers, modifying their dynamics and thus to act indirectly on the mechanical properties of the cytoskeletal and cellular physiology [11, 12]. Actin cytoskeletal dynamics play a crucial role in neuronal system development and drive important processes such as neurite extension, formation of axon, dendrites and growth cones migration [13, 14]. Fath and colleagues [15] demonstrated, for the first time, that TMOD1 and TMOD2 are negative regulators of neurite outgrowth and they have a key role in neurites formation and extension. TMODs can stabilize actin filaments that are not available for the formation of new neurites, or reduce the actin levels required for the polymerization. TMODs are also able to influence positively dendritic arbor, indeed overexpression of both TMOD1 and TMOD2 increased dendritic complexity and the branching [16]. Recently, TMODs are emerging as new protagonists in several diseases pathogenesis; TMOD2 has altered expression in fetal Down syndrome [17], mesial temporal lobe epilepsy [18], post-stroke [19] and post-methamphetamine exposure [20], while TMOD3 has been reported as a novel biomarker with high sensitivity and diagnostic accuracy in endometriosis [21]. Initial studies reported TMODs role in cancer; Kureha and colleagues [22] demonstrated that TMOD1 expression was directly regulated by NF-κB and its overexpression was associated with enhanced breast tumor growth in a mouse xenograft model. TMOD1 is a powerful diagnostic marker for ALK-negative anaplastic large-cell lymphoma [23] and it was overexpressed frequently in oral squamous cell carcinoma [24]. TMOD2 high expression levels correlate with high survival probability and favorable disease outcome in neuroblastoma patients’ [25], while TMOD3 up regulation is responsible for chemotherapeutic agents’ resistance in non-small cell lung carcinoma [26]. Our data clearly demonstrated extensively a positive and specific correlation between TMOD1 and TMOD2 expression levels and neuroblastoma; high expression levels of these two genes were associated with high survival probability and good prognosis of neuroblastoma patients’. *In vitro* characterization and functional studies led us to better understand the role of TMOD1 in neuroblastoma cell lines. In particular, we identified that TMOD1 knockin caused cell cycle arrest, cell proliferation arrest in addition to a functional and mature cell differentiation. On the contrary, TMOD1 knockdown induced loss of expression of mature neuronal markers and the production of an unfavorable cell differentiation profile.

## RESULTS

### *TMOD1* and *TMOD2* high expression levels are associated with high survival probability neuroblastoma patients’

**Figure 1:**
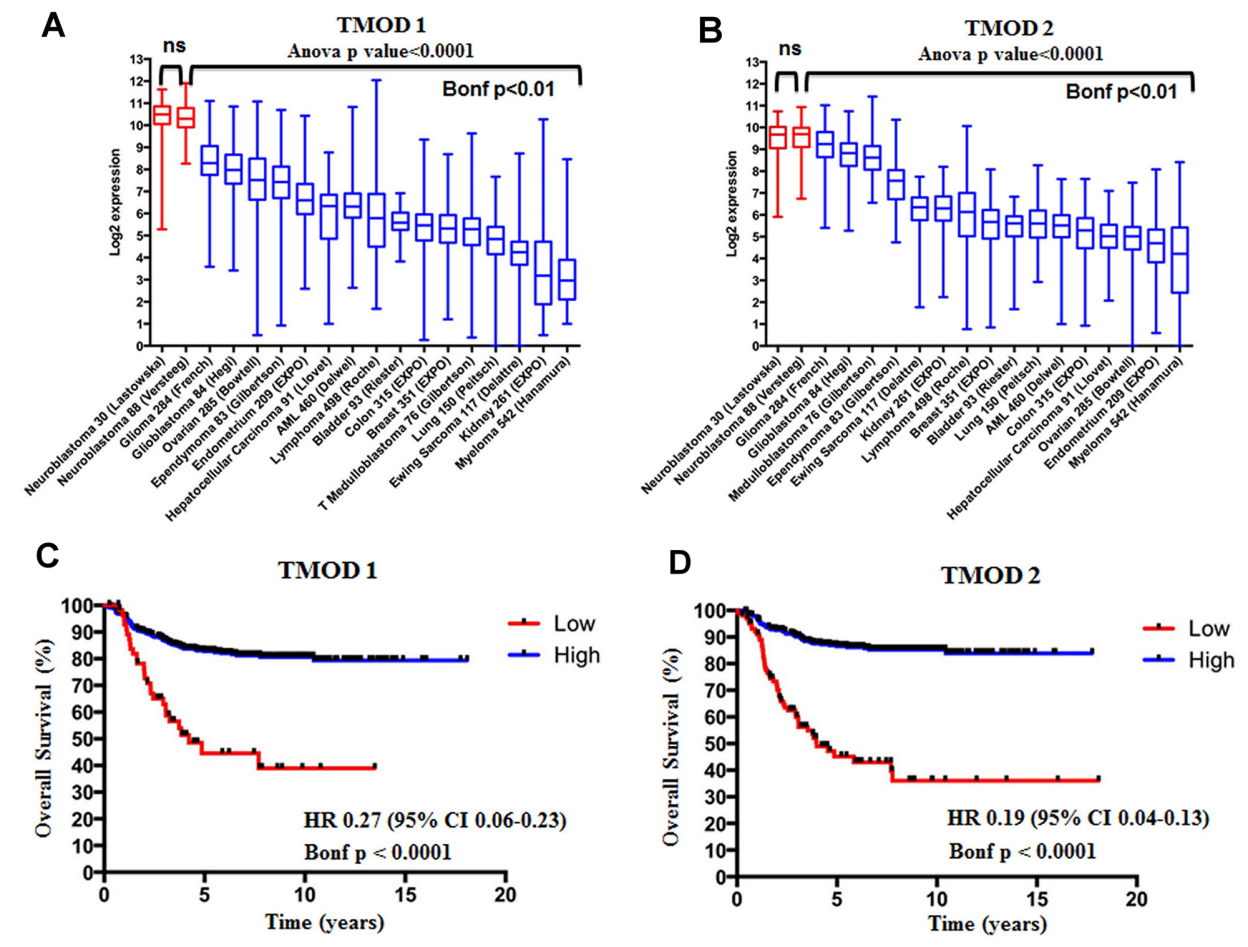
*TMOD1* and *TMOD2* genes are more expressed in neuroblastoma than in other tumors types and their high expression levels correlate with a good overall survival The box and whisker plots show the expression of *TMOD1* **(A)** and *TMOD2* **(B)** in 18 different data sets. Red and blue boxes and whiskers refer to the neuroblastoma and other tumor types datasets, respectively. The box and whisker are sorted by decreasing order of median expression value. Plots are entitled with ANOVA p value and the corresponding gene symbol. The Bonferroni (Bonf) adjusted p value obtained by post-hoc analysis is reported below the black bar. We considered significant a p value<0.05. Kaplan-Meier curves show overall survival in the 498 patients’ cohort stratified by *TMOD1* **(C)** and *TMOD2* **(D)**. Plots are entitled with the corresponding gene symbol. High expression (blue) and low expression (red) curves were compared by log-rank test and corrected for multiple hypotheses testing by Bonferroni method. Each plot reports the Bonferroni corrected p value (Bonf), the hazard ratio and confidence interval of the stratification. HR: hazard ratio. CI: confidence interval. ns: not significant.

**Figure 2:**
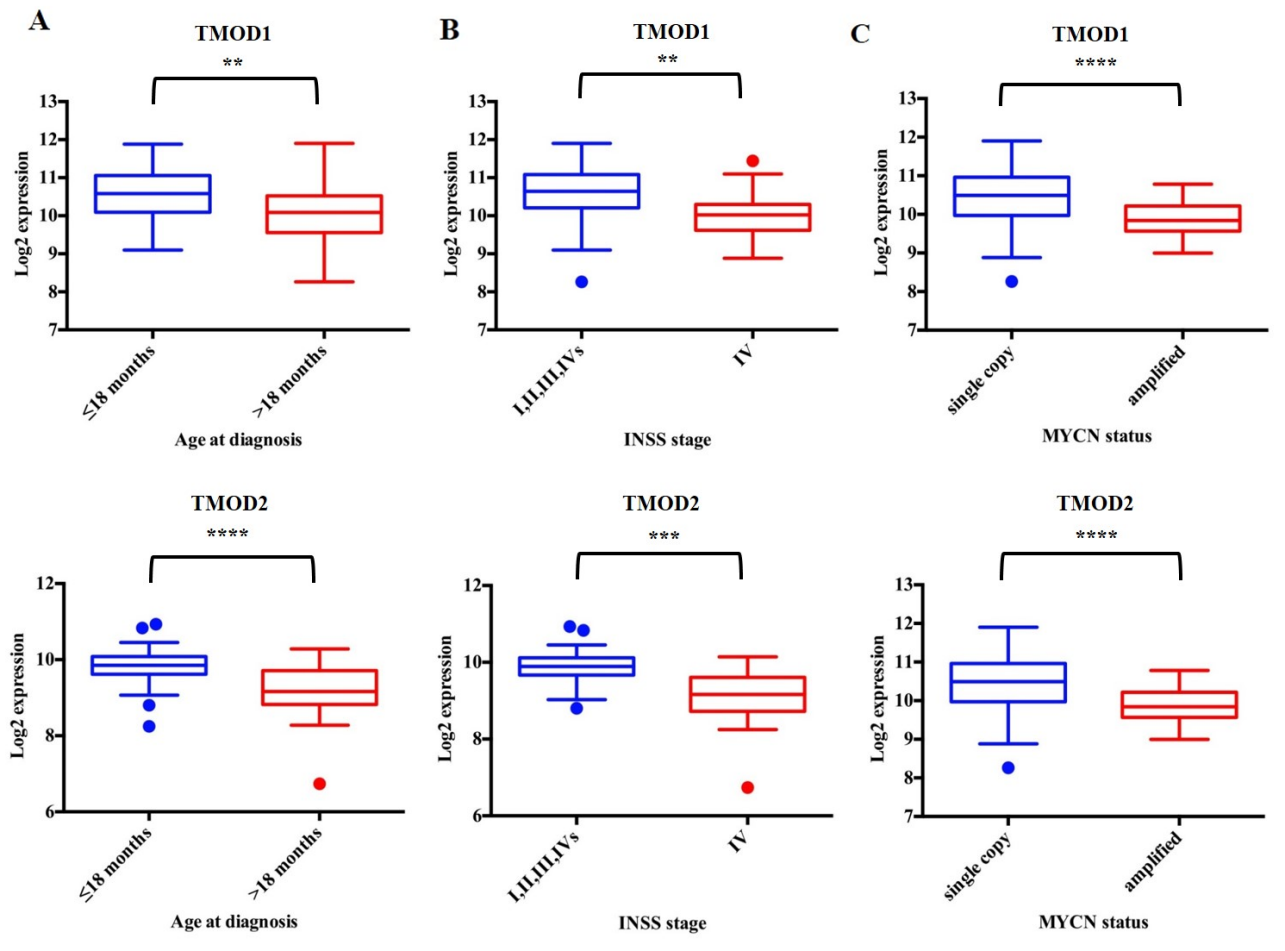
*TMOD1* and *TMOD2* expression levels correlate with favorable clinical and molecular characteristics Box and whisker plots of *TMOD1* and *TMOD2* in the populations defined by Age at diagnosis **(A)**, INSS stage **(B)** and *MYCN* status **(C)** risk factors. Data are relative to the patients in the 88 patients’ cohort. Plots are entitled with the corresponding gene symbol. Significance of the difference between the means has been assessed by unpaired *t* test. A probability smaller than 0.05 was considered a significant difference. ^**^ means p<0.001, ^***^ means p<0.0001 and ^****^ means p<0.00001. Age at diagnosis (≤ 18 months (blue) vs. > 18 months (red)), INSS stage (I, II, III, IVs (blue) vs. IV(red)) and *MYCN* status (single copy (blue) vs. amplified (red)) groups define the clinical and molecular sub-populations.

We assessed the expression of *TMOD1* and *TMOD2* genes in 17 different datasets comprising different types of tumors and we added an additional neuroblastoma dataset as control (Supplementary Table 1). We found that *TMODs* expression varied among tumor types but the highest expression of *TMOD1* (Figure 1A) and *TMOD2* (Figure 1B) was observed in neuroblastoma tumors (Bonf p<0.01). We have not found a significant difference of expression for either genes between the two neuroblastoma datasets supporting the conclusion that either genes are highly expressed in neuroblastoma (p>0.05, Figure 1A and 1B). These findings raised the question of the prognostic significance of these genes. We studied the survival curves of 498 neuroblastoma patients split in high or low *TMODs* expression. We found that 440 patients with high *TMOD1* expression had a good overall survival (5y-OS±SE: 0.82±0.02%) as opposed to 58 patients with low *TMOD1* expression levels who had a poor overall survival (5y-OS±SE: 0.44±0.07) (Figure 1C). Likewise, 397 patients with high expression levels of *TMOD2* had a good overall survival (5y-OS±SE: 0.86±0.01) as opposed to 101 patients with low *TMOD2* expression levels who had a poor overall survival (5y-OS±SE: 0.45±0.05%) (Figure 1D). Overall survival between low and high expression of *TMODs* was significantly different (*TMOD1* HR 0.27 95%CI 0.06-0.23 and *TMOD2* HR 0.19 95%CI 0.04-0.13 both Bonf p<0.0001). These results demonstrated that *TMODs* were prognostic and that good prognosis was associated with high expression levels. We confirmed the observation that *TMOD1* and *TMOD2* stratified patients into groups with different overall survival (*TMOD1* HR 0.15 95%CI 0.02-0.14, and *TMOD2* HR 0.06 95% CI 0.02-0.1, both Bonf p<0.0001 and TMOD1 HR 0.2 95% CI 0.01-0.2, and TMOD2 HR 0.2 95% CI 0.06-0.3, both Bonf p<0.0001), and that high expression levels of *TMOD1* and *TMOD2* were associated with good prognosis on two independent datasets of 88 neuroblastoma primary tumors profiled by Affymetrix platform and 251 neuroblastoma primary tumors profiled by customized 11K oligonucleotide-microarrays (Supplementary Figure 1). Analysis of the relationship between *TMODs* expression and known neuroblastoma risk factors indicated that *TMODs* maintained a significant prognostic value when the model was adjusted for the clinical and molecular covariates (*TMOD1* OS HR 0.4 95% CI 0.2-0.7 and *TMOD2* HR 0.5; 95% CI 0.2-0.8, both p≤0.01) (Supplementary Table 2) demonstrating that *TMODs* expression is an independent risk factors for neuroblastoma patients. Accordingly, *TMOD1* and *TMOD2* expression was significantly higher in low risk than in high risk patients. Higher *TMODs* expression was observed in patients with less than 18 months of age at diagnosis (p<0.01; Figure 2A), in stage I, II, III, IVs tumors (p<0.01; Figure 2B) and in *MYCN* single copy tumors (p<0.01; Figure 2C). These findings were confirmed in an independent dataset (Supplementary Figure 2) supporting the conclusion that *TMODs* expression is elevated in patients with favorable clinical and molecular characteristics.

### TMODs have different expression levels and cellular localization in differentiated neuroblastoma cells

**Figure 3:**
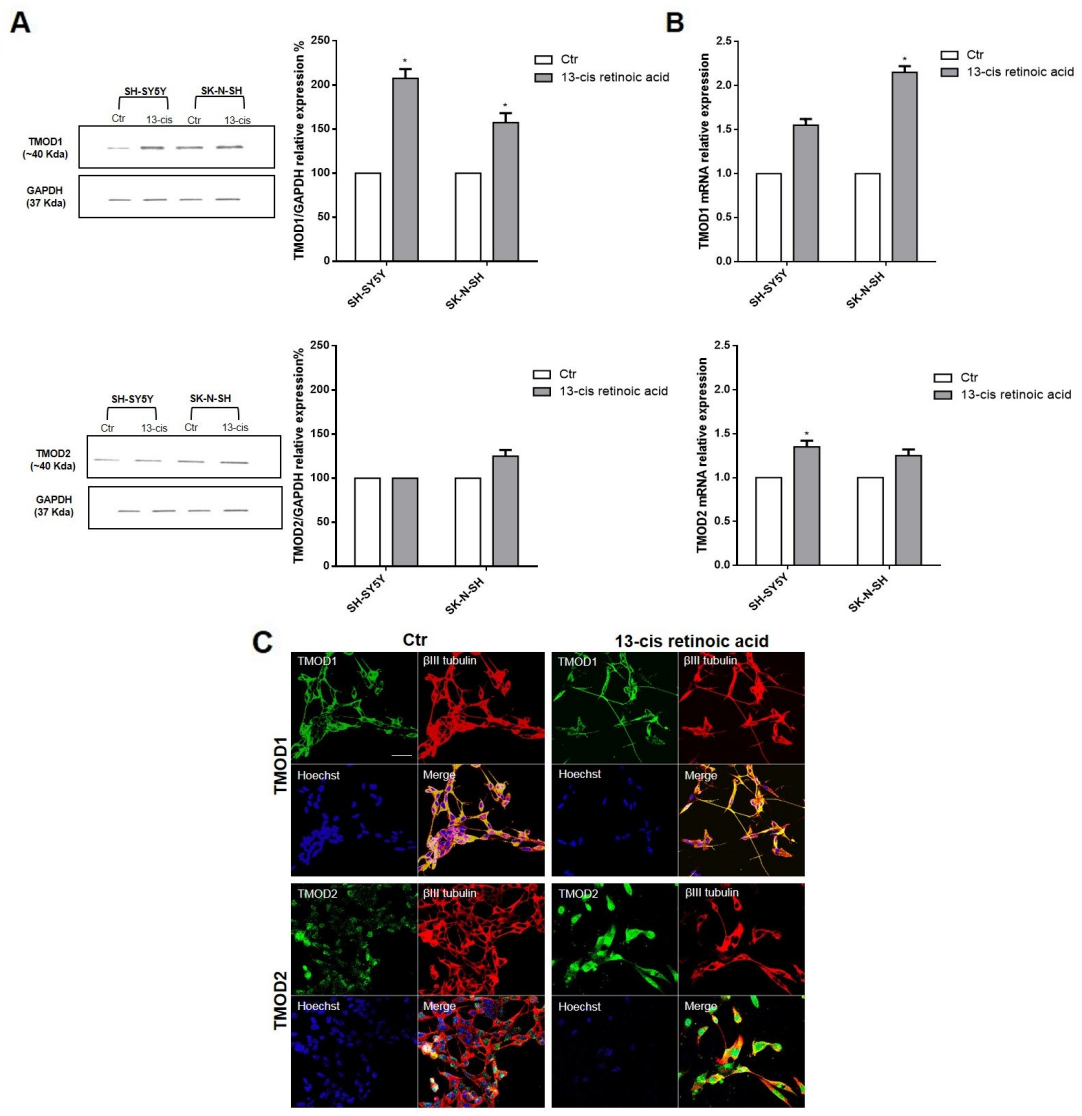
TMOD1 and TMOD2 have different expression levels and cellular localization in neuroblastoma cell lines **(A)** Western Blotting analysis of TMOD1 and TMOD2 protein expression levels in SH-SY5Y and SK-N-SH untreated and 13-cis retinoic acid treated cells. GAPDH was used as a loading control. Densitometric analysis of TMOD1 and TMOD2 protein expression levels was obtained using Image Studio™ Software- LI-COR System. **(B)** qRT-CR analysis of TMOD1 and TMOD2 mRNA expression levels in SH-SY5Y and SK-N-SH untreated and 13-cis retinoic acid treated cells. Data are normalized to the GAPDH signal and are obtained from three experiments. ^*^ means p<0.05 vs. control. **(C)** Representative confocal images of SH-SY5Y neuroblastoma cells after 72 hours in culture medium alone (Ctr) or in medium added 40 μM 13-cis retinoic acid. TMOD1 and TMOD2 antibodies were in green, β III tubulin antibody was in red and Hoechst, used as nuclei marker, was in blue. Merge represents the superposition of the three signals. Scale bar, 40 μm.

Neuroblastoma patients’ data identified a positive correlation among *TMODs* high expression levels, good survival probability and favorable prognosis. On this basis, we investigated the role of TMODs *in vitro* on the biology of neuroblastoma cell lines. First, we evaluated the presence and the expression levels of TMOD1 and TMOD2 in SH-SY5Y and SK-N-SH neuroblastoma cells. Proteins and mRNA expression levels were studied in untreated and 13-cis retinoic acid treated cells, currently used in neuroblastoma therapy as cell-differentiating agent in association with standard therapeutic protocols [27]. Neuroblastoma cells were cultured for 72 hours after 13-cis retinoic acid treatment and then analyzed by Western Blotting and qRT-PCR. We observed, in both cell lines, that TMOD1 expression was barely detectable in the control group and significantly increased after 13-cis retinoic acid treatment; instead, TMOD2 protein expression level was present in untreated cells and did not increase after 13-cis retinoic acid treatment (Figure 3A, 3B). These results are in agreement with the study of Fath and colleagues [15] in N2a neuroblastoma cells. To better-characterize TMODs behaviour, we studied the localization of these two proteins in neuroblastoma cell lines by immunocytochemistry. Staining TMODs in both cell lines, it emerged that TMOD1 has a cytoplasmic localization and it is expressed all along the cell neurites, while TMOD2 has a mainly perinuclear localization (Figure 3C).

### TMOD1 knockin induces cell cycle arrest in G0/G1 phase and cell proliferation arrest

**Figure 4:**
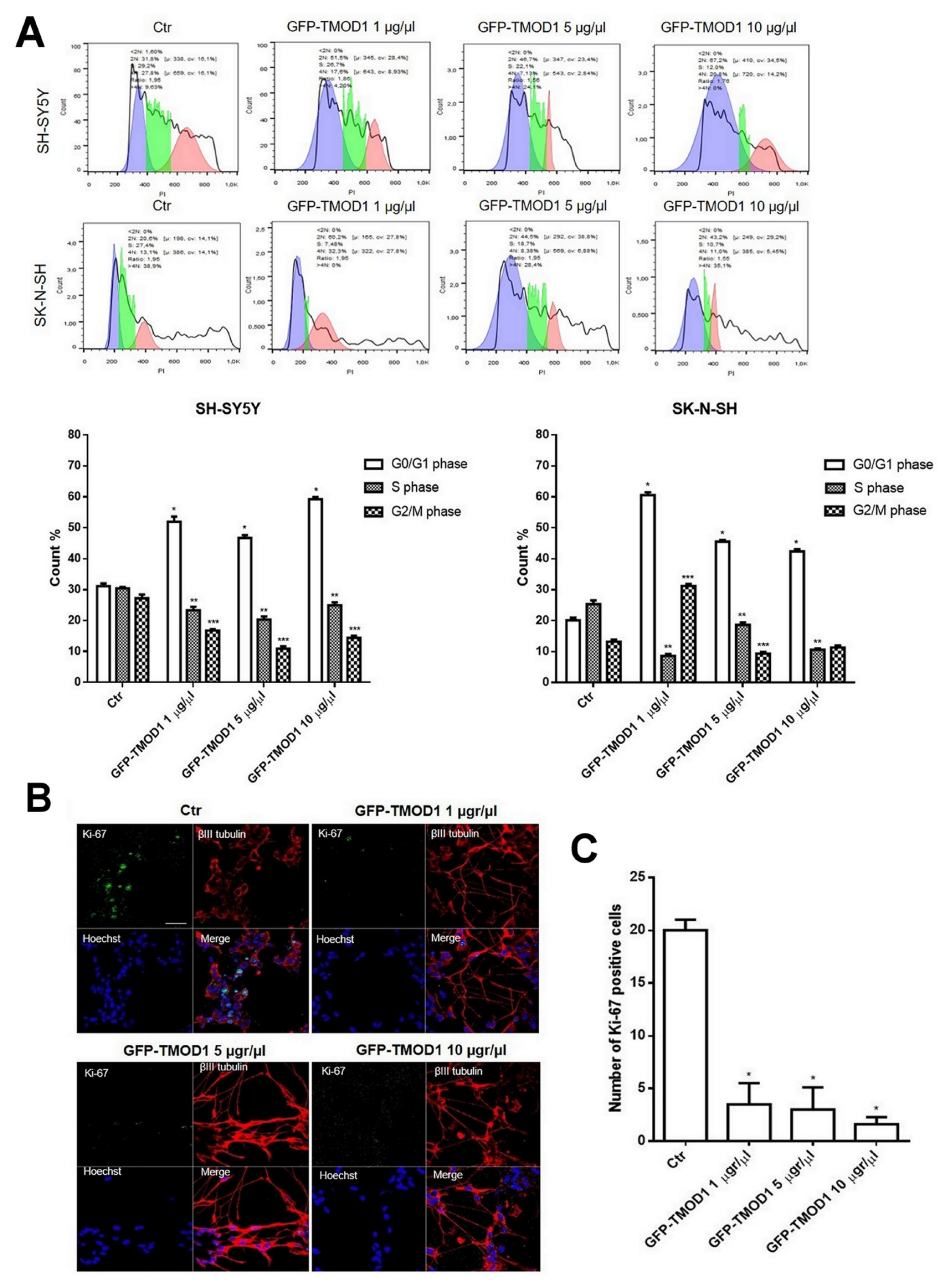
TMOD1 knockin induces cell cycle and cell proliferation arrest **(A)** Cell cycle analysis of SH-SY5Y and SK-N-SH neuroblastoma cells transfected with *GFP-TMOD1* plasmid for 72 hours with three different concentrations (1 μg/μl, 5 μg/μl, 10 μg/μl). ^*^means p<0.0001 vs. G0/G1 phase control; ^**^ means p<0.0001 vs. S phase control; ^***^ means p<0.0001 vs. G2/M phase control. Data are obtained from three experiments. **(B)** Representative confocal images of SH-SY5Y neuroblastoma cells transfected with *GFP-TMOD1* plasmid for 72 hours. Ki-67, proliferation marker, was in green, β III tubulin antibody was in red and Hoechst, used as nuclei marker, was in blue. Merge represents the superposition of the three signals. Scale bar, 40 μm. **(C)** Quantitative analysis of proliferation cells was determined by counted the number of Ki-67 nuclei positive cells at least 10 fields for each treatment. ^*^ means p<0.001 vs. control.

Since both TMODs correlate positively with neuroblastoma and only TMOD1 expression levels increased significantly after 13-cis retinoic acid treatment, we decided to investigate the effects of TMOD1 overexpression in neuroblastoma cell lines measured by cell cycle, proliferation and cell differentiation parameters. For cell cycle analysis, neuroblastoma cell lines were transfected with *GFP-TMOD1* plasmid for 72 hours at three different concentrations (1 μg/μl, 5 μg/μl, 10 μg/μl). Flow cytometry analysis showed that TMOD1 knockin induced cell cycle arrest in G0/G1 phase (Figure 4A). In particular, there was a significant increase in the cells number in G0/G1 phase and a decrease of cells number in S phase cell cycle, compared to control. The data indicated that, in both cell lines, TMOD1 knockin blocked cells in G0/G1 phase decreasing the tumor cells number in proliferation. The cell proliferation arrest was evaluated also by immunocytochemistry using cell proliferation marker Ki-67; TMOD1 knockin induced a significant cell proliferation arrest, in a dose dependent manner, with a decrease in the number of Ki-67 nuclei positive cells compared to the control (Figure 4B). A quantitative analysis of cell proliferation arrest was obtained by counting the number of Ki-67 nuclei positive cells in a minimum of 10 fields for each treatment (Figure 4C). A direct nuclei cell count was also performed confirming a significant reduction of cells number induced by TMOD1 knockin compared to the control (mean cell number/field ± S.E.M.: 70.0 ± 8.375 for control, 48.38 ± 3.343 for TMOD1 1μg/μl, 45.80 ± 6.996 for TMOD1 5 μg/μl, 29.80 ± 6.689 for TMOD1 10 μg/μl).

### TMOD1 knockin induces in neuroblastoma cells a neuron-like differentiated profile

**Figure 5:**
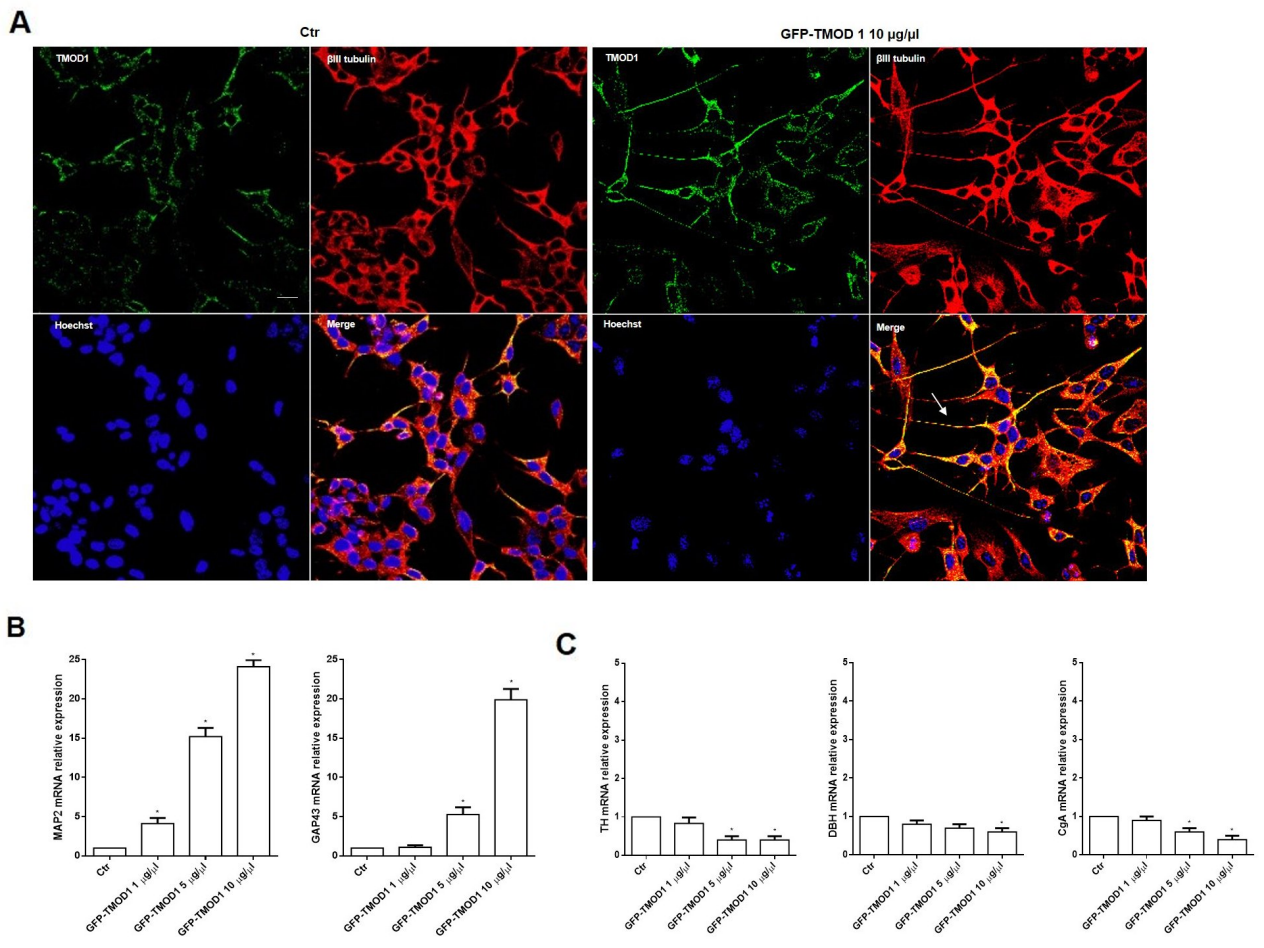
TMOD1 knockin induces in neuroblastoma cells a neuron-like differentiated profile **(A)** Representative confocal images of SH-SY5Y neuroblastoma cells transfected with *GFP-TMOD1* plasmid at the concentration of 10 μg/μl for 72 hours. TMOD1 was in green, β III tubulin antibody was in red and Hoechst, used as nuclei marker, was in blue. Merge represents the superposition of the three signals. Arrow indicates neurite branching and varicosities. Scale bar, 40 μm. **(B)** qRT-PCR analysis of MAP2 and GAP43 mRNA expression levels in SH-SY5Y untreated and *GFP-TMOD1* plasmid transfected cells. **(C)** qRT-PCR analysis of TH, DBH and CgA mRNA expression levels in SH-SY5Y untreated and *GFP-TMOD1* plasmid transfected cells. Data are normalized to the GAPDH signal and are obtained from three experiments. ^*^ means p<0.05 vs. control.

Cells differentiation was analyzed in both cell lines by immunocytochemistry and representative images by confocal microscopy showed that TMOD1 knockin induced neuroblastoma cells differentiation characterized by long neurites with sprouting, growth cones and varicosities (Figure 5A). Neurites arborization and the formation of varicosities are the main features of functional and mature cell differentiation [28–30]. It was investigated in detail the biochemical implications of cell differentiation induced by TMOD1 knockin by evaluating the expression levels of a set of differentiation markers. qRT-PCR analysis showed a significant increase of neuronal markers such as Microtubule-associated protein 2 (MAP2) and Growth Associated protein 43 (GAP43) (Figure 5B), while Tyrosine hydroxylase (TH), Dopamine beta-hydroxylase (DBH) and Chromogranin A (CgA) showed a decrease in mRNA expression levels (Figure 5C). MAP2 and GAP43 proteins are involved in neurogenesis and their expression is indicative of neuronal growth, development and stabilization. In conclusion, the morphological and biochemical data collected suggested that TMOD1 overexpression induced the development of a neuronal cell differentiation profile.

### TMOD1 knockdown induces in neuroblastoma cells a neuroendocrine differentiation profile

**Figure 6:**
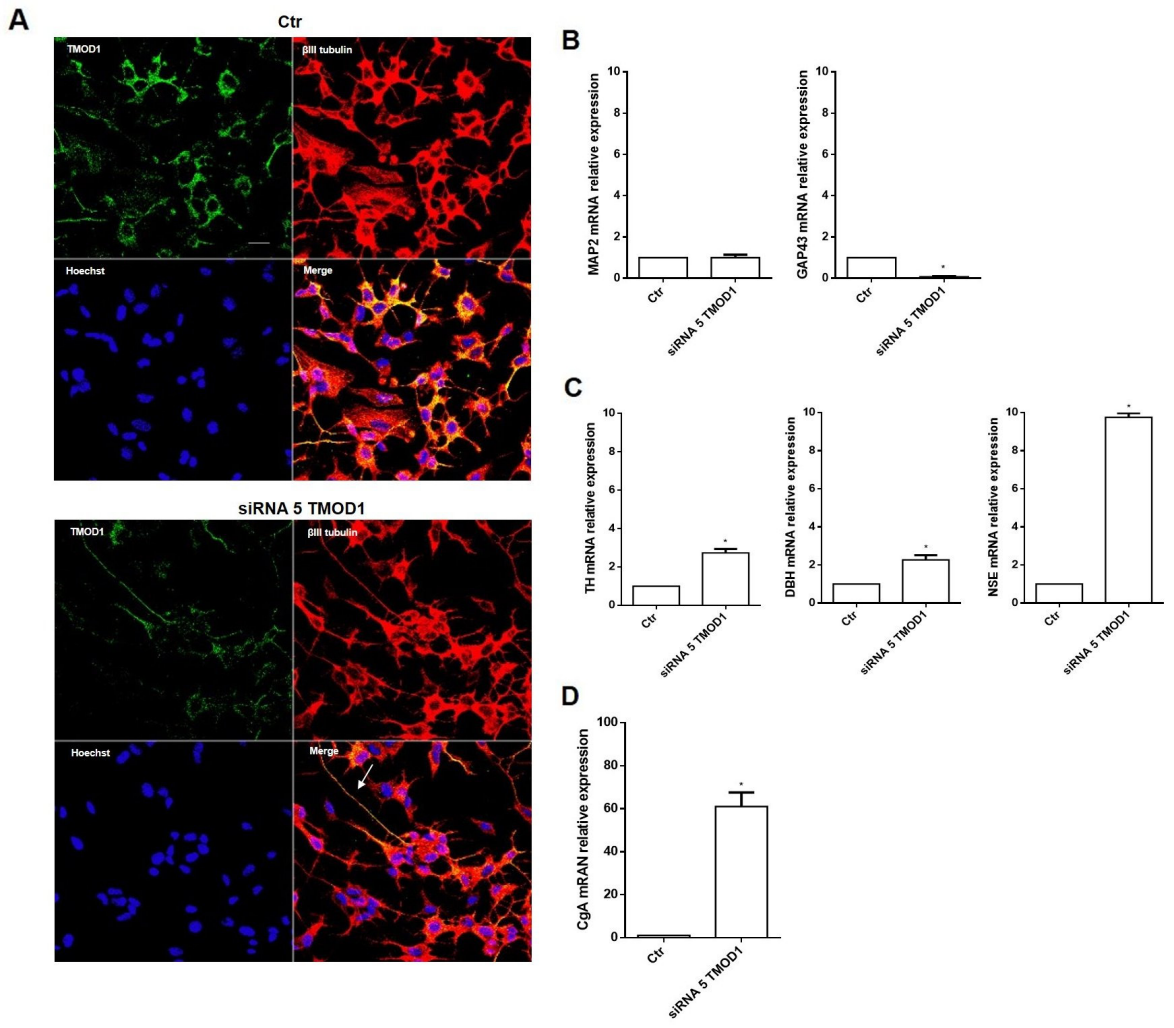
TMOD1 knockdown induces in neuroblastoma cells a neuroendocrine differentiation profile **(A)** Representative confocal images of siRNA 5 TMOD1 downregulation effects in SH-SY5Y neuroblastoma cells after 72 hours of transfection. TMOD1 was in green, β III tubulin antibody was in red and Hoechst, used as nuclei marker, was in blue. Merge represents the superposition of the three signals. Arrow indicates thin neurite without varicosities. Scale bar, 40 μm. **(B)** qRT-PCR analysis of MAP2 and GAP43 mRNA expression levels in SH-SY5Y untreated and TMOD1 knockdown treated cells. **(C)** qRT-PCR analysis of TH, DBH and NSE mRNA expression levels in SH-SY5Y untreated and TMOD1 knockdown treated cells. **(D)** qRT-PCR analysis of CgA mRNA expression levels in SH-SY5Y untreated and TMOD1 knockdown treated cells. Data are normalized to the GAPDH signal and are obtained from three experiments. ^*^ means p<0.05 vs. control.

To further examine the TMOD1 functional role in neuroblastoma cells, we studied the effects of TMOD1 downregulation utilizing RNA interference technique. TMOD1 downregulation, as occur with TMOD1 overexpression, induced both cell cycle and proliferation arrest (data not shown), but surprisingly it induced a new differentiation profile characterized by long and thin neurites, without sprouting, growth cones and varicosities (Figure 6A). The cell differentiation observed was very different compared to that obtained after TMOD1 overexpression. To better understand the nature of this type of differentiation, we evaluated the expression of a set of differentiation markers by qRT-PCR. After TMOD1 knockdown, Microtubule-associated protein 2 (MAP2) mRNA expression levels remained unchanged while there was a decrease of Growth Associated protein 43 (GAP43) neuronal marker (Figure 6B). Conversely, a significant increase of Tyrosine hydroxylase (TH), Dopamine beta-hydroxylase (DBH), and Neuron-specific enolase (NSE) was observed (Figure 6C). In particular, we observed a significant increase of Chromogranin A (CgA) mRNA expression levels (Figure 6D), a neuroendocrine secretory protein, which is a useful prognostic and diagnostic tool for neuroblastoma and correlates with tumor burden and survival patients’ [31]. These data suggest that TMOD1 downregulation induced the loss of neuron-like differentiated profile towards neuroendocrine profile.

## DISCUSSION

The understanding of biological mechanisms and biomarkers involved in the onset and progression of neuroblastoma is needed to reach better prognostic stratification and more efficacious therapeutic strategies. In the present study, we demonstrated that *TMOD1* and *TMOD2* expression are independent prognostic markers and that high expression levels of these genes are associated with favorable outcome. Little is known about the relationship between neuroblastoma and *TMOD* genes. *TMOD2* is part of a 160 gene signatures predicting neuroblastoma outcome [32] and of a 55 gene signature predicting neuroblastoma outcome in metastatic neuroblastoma lacking *MYCN* amplification [33]. There is no evidence of studies on *TMOD1* and neuroblastoma. *TMOD1* and *TMOD2* were highly expressed in neuroblastoma, relative to other tumor types, suggesting an important role of these genes in the tumor homeostasis. Moreover, both genes showed a strong association between high expression and favorable neuroblastoma prognosis. The strength of this result relies on the large number of samples in the three neuroblastoma patients’ datasets (837 tumor samples) that have been used to reach this conclusion; this conclusion was further supported by the observation that newborn patients, no *MYCN* amplification, or low grade stage had a higher expression of *TMOD*1 and *TMOD*2 in two independent datasets. *In vitro* functional studies demonstrated that TMOD1 knockin induced cell cycle arrest in G0/G1 phase, cell proliferation arrest, a functional and mature neuron-like differentiation. The biochemical differentiation profile of neuroblastoma cells overexpressing TMODs showed a significant increase of MAP2 and GAP43, mimicking then the “neuronal differentiation” obtained by retinoic acid, and an overall decrease of TH, DBH and CgA mRNA expression levels. On the contrary, TMOD1 downregulation caused the loss of mature and functional differentiation cell profile, with the appearance of long and thin neurites without sprouting and varicosities, the accumulation sites of the secretory vesicles of neurotransmitters that are released in response to neurosecretory stimuli [29, 30]. Biochemical analysis evidenced a decrease of neuronal biomarkers expression and a significant increase of TH, DBH, NSE and CgA, indexes of neuroendocrine differentiation and considered unfavorable prognostic neuroblastoma biomarkers [34]. In a recent study, Lee and colleagues [35] evaluated the clinical significant of TH mRNA transcripts levels in the peripheral blood of patients with neuroblastoma demonstrating that TH expression was associated with high-risk features, advanced stage and worse outcome. Previous studies also reported that positive TH expression was a poor prognostic indicator in metastatic neuroblastoma [36, 37], suggesting a role of this marker in stratification. In particular we observed an increase of NSE by tenfold and CgA mRNA expression sixty-fold higher than control. CgA is a pro-hormone that is stored in the same vesicles and co-released with catecholamines in adrenal medulla and postganglionic sympathetic axons and regulates in turn chatecolamines secretion with a negative feedback mechanism [38]. NSE and CgA expression correlate with the differentiation level of the tumor, metastatic sites and response to treatment; patients in stage I and stage II have low levels of CgA and favorable outcome, while patients with advanced disease stages (III and IV) have high levels of NSE and CgA and bad prognosis [39, 40]. Furthermore hypoxia, validated as predictor of poor outcome in neuroblastoma, was found to induce a shift from neuronal to chromaffin cell differentiation, with high expression of neuroendocrine markers and decrease in neuronal markers as GAP43 [41]. Finally, GSK inhibitors were found to arrest neuroblastoma cells proliferation coupled with a decrease in neuroendocrine markers [42]. Thus, in our experimental data, TMOD1 knockdown induced high expression levels of all unfavorable neuroendocrine biomarkers, delineating an aggressive tumor profile. In conclusion, TMODs are able to influence positively survival probability and prognosis of neuroblastoma patients mainly acting on cell differentiation. Sympathetic neurons and chromaffin cells derive from a common fate-restricted sympathoadrenal progenitor of the neural crest, which, in response to the local environment and a network of transcription factors, can choose one or the other differentiation path [43]. Thus, TMODs could represents, in prenatal and early post-natal development, a signal for the correct differentiation of sympathetic cells. Although further investigation is required, these data identify a new pathway involved in neuroblastoma and therefore new strategies will develop to tackle the tumor.

## MATERIALS AND METHODS

### Gene expression datasets

Three publically available datasets were used for gene expression analysis. The first dataset [44] contains the gene expression profile of 498 neuroblastomas measured by the Illumina HiSeq 2000 RNAseq platform (GSE62564). The second one [45] contains the gene expression profile of 88 tumors measured by the Affymetrix Human Genome U133 Plus 2.0 platform (GSE16476). The third one [Customized oligonucleotide microarray gene expression-based classification of neuroblastoma patients outperforms current clinical risk stratification. Journal of Clinical Oncology, 24, 5070–5078] contains the gene expression profile of 251 tumors generated as dye-flipped dual-color replicates using customized 11K oligonucleotide-microarrays (E-TABM-38). In addition, we utilized 3929 publically available expression profiles from 17 different tumor types by the Affymetrix Human Genome U133 Plus 2.0 platform and stored in the R2: Genomic Analysis and Visualization Platform (http://r2.amc.nl). Good and poor outcome were defined as the patient’s alive or dead status 5 years after diagnosis.

### Cell culture

The human SH-SY5Y neuroblastoma cell line was cultured in a 1:1 mixture of Ham’s F12 nutrient and Dulbecco’s modified Eagle’s medium (Sigma-Aldrich) supplemented with 10% fetal bovine serum (FBS, Sigma-Aldrich), 2 mM L-glutamine, 50 mg/mL penicillin, and 100 mg/mL streptomycin (Sigma-Aldrich). The SK-N-SH neuroblastoma cell line was cultured in Dulbecco’s modified Eagle’s medium (Sigma-Aldrich) supplemented with 10% fetal bovine serum (FBS, Sigma-Aldrich), 2 mM L-glutamine, 50 mg/mL penicillin, and 100 mg/mL streptomycin (Sigma-Aldrich). All the cell lines were grown at 37°C in a 95% air–5% CO_2_ humidified incubator. Cell lines, at passage lower than 10, were used within 3 months after receipt. Cells supplied by ECACC, the European Collection of Authenticated Cell Cultures, undergo quality control and authentication procedures. These include testing for mycoplasma by culture isolation, Hoechst DNA staining and PCR, together with culture testing for contaminant bacteria, yeast and fungi. Authentication procedures used include species verification by DNA barcoding and identity verification by DNA profiling. Human cell lines are analyzed by PCR of short tandem repeat sequences within chromosomal microsatellite DNA (STR-PCR).

### 13-cis Retinoic acid treatment

Twenty-four hours after seeding in complete media, neuroblastoma cell lines were cultured with 40 μM 13-cis retinoic acid (Sigma-Aldrich). At 80% confluence, after 72 hours, cells were collected and proteins were extracted for Western Blotting analysis or fixed for immunofluorescence and morphologic analysis.

### DNA plasmid amplification and transient transfection

*GFP-TMOD1* plasmid was processed with bacterial transformation and amplification. The *GFP-TMOD1* plasmid was purified by Plasmid Midiprep Kit (Sigma-Aldrich), according to the manufacturer’s protocol. SH-SY5Y and SK-N-SH neuroblastoma cell lines were transfected with the plasmid, at three different concentrations, using Lipofectamine reagent 2000 (Invitrogen). The complete process is indicated in Supplementary Materials and Methods.

### siRNA transient transfection

The human-specific *TMOD1* interference was performed assessing the efficacy of four different siRNAs at different concentrations to evaluate which siRNA was able to induce a greater downregulation of TMOD1 mRNA expression levels (Supplementary Figure 3). SH-SY5Y and SK-N-SH neuroblastoma cell lines were transfected with siRNA 5 at the concentration of 10 nM using Hi-perfect transfection reagent (Qiagen) in culture medium 1 day after seeding. Cells were cultured for 72 hours after transfection with siRNA 5 and then collected for the mRNA expression levels analysis.

### RNA extraction and quantitative RT- PCR

The total RNA was isolated from SH-SY5Y and SK-N-SH neuroblastoma cells using the RNeasy kit (Qiagen) and digested with the RNase-Free DNase set (Qiagen), according to the manufacturer’s protocol. RNA was retrotranscribed using murine leukemia virus reverse transcriptase (Promega Italia) and oligo (dT) 15-18 as a primer. Quantitative RT-PCR (qRT-PCR) was performed by the SYBR Green I system (BioRad Italy) and detection were performed with the ViiA7™ RT-PCR System (Applied Biosystems). Primers and complete protocol are indicated in Supplementary Materials and Methods.

### Western blotting

Total cell lysates were prepared by scraping the cells in lysis buffer. 15 μg of total proteins were electrophoresed into 10% SDS-PAGE precast gel, transferred to nitrocellulose paper, and stained with the antibodies of interest, followed by the appropriate secondary antibodies. Antibodies and full protocols are listed in Supplementary Materials and Methods.

### Immunofluorescence analysis

Neuroblastoma cells were plated with a density of 75 × 10^3^/well in a 24 wells plate, fixed in ice-cold methanol, washed and incubated in Phosphate Buffered Saline and Bovine Serum Albumin. Cells were stained with the primary antibodies of interest, overnight at 4°C, followed by the secondary antibodies conjugated with Alexa Fluor^®^ 488 or CY™3. Antibodies and full protocols are listed in Supplementary Materials and Methods.

### Flow cytometry analysis

For cell cycle analysis, cells were harvested at the completion of the siRNA 5 TMOD1 and *GFP-TMOD1* transfections and washed with phosphate-buffered saline (PBS; pH 7.4) before being fixed with 70% ethanol on the wheel for 15 minutes at 4°C. Subsequently, the cells were centrifuged at 4500 rpm for 5 minutes at 4°C, washed with phosphate-buffered saline and cells were resuspended in 600 μl of 0,1% sodium citrate (Sigma-Aldrich), 50 μg/ml of propidium iodide (PI, Sigma-Aldrich) and 10 μg/ml of Ribonuclease A (Sigma-Aldrich) for staining cellular DNA. The cellular DNA content was then analyzed using a MACS Quant Flow Cytometer (Miltenyi Biotech). Data analysis was carried out using FlowJo software V10.

### Bioinformatics and statistical analysis

Log2-transformed gene expression values were utilized whenever possible. Gene symbol was selected as reference annotation. In the Affymetrix platform the highest probe set expression was associated to the gene symbol [45]. Analysis was carried out, in part by the R2: Genomic Analysis and Visualization Platform (http://r2.amc.nl). Patients overall survival (OS) was assessed by Kaplan-Meier curves. Survival probability was reported as 5-years overall survival ± standard error (5y-OS±SE). Log-rank test, corrected for multiple hypotheses testing by the Bonferroni method, assessed the significance of the separation between survival curves. Multivariate analysis with a Cox proportional regression model evaluated the relationship among prognostic factors. The validity of the proportional hazards assumption was tested via evaluation of scaled Schoenfeld residuals. The significance of the difference between mean values was determined by paired or unpaired *t*-test or by ANOVA Kruskal-Wallis test when the comparison involved more than two groups with significantly different standard deviation.

## SUPPLEMENTARY MATERIALS FIGURES AND TABLES






