# Supplementary material for: Favorable prognostic role of tropomodulins in neuroblastoma
Source: Oncotarget. 2018 Jun 5;9(43):27092–103. doi: 10.18632/oncotarget.25491 (PMC6007461; doi:10.18632/oncotarget.25491)
Supplement: Supplementary file 1 [file oncotarget-09-27092-s001.pdf]

Favorable prognostic role of tropomodulins in neuroblastoma

SUPPLEMENTARY MATERIALS AND METHODS

DNA plasmid amplification and transient transfection

GFP-TMOD1 plasmid was sent by Prof. Velia M. Fowler of the Scripps Research Institute of California and it was processed with bacterial transformation and amplification, as follows. The construct was eluted from paper and mixed with TOP10 competent cells (Invitrogen) for a heat-shock transformation. The transformed cell suspension was grown in a pre-warmed SOC medium (Invitrogen) for 1 hour at 37°C and then it was spread on LB agar plates that were incubated at 37°C overnight. Positive colonies were picked and inoculated in LB broth, with the appropriate antibiotic, and incubated at 37°C for 8 hours. Than colonies were incubated in higher amount of LB medium with ampicillin at 37°C for 12-16 hours and the absorbance, to calculate the optimal volume of culture to obtain a good plasmid yields, was read. The plasmid was subjected to purification by Plasmid Midiprep Kit (Sigma-Aldrich), according to the manufacturer's protocol. SH-SY5Y and SK-N-SH neuroblastoma cell lines were transfected with *GFP-TMOD1* plasmid, at three different concentrations (1 µg/µl, 5 µg/µl, 10 µg/µl), using Lipofectamine reagent 2000 (Invitrogen). Plasmid and Lipofectamine reagents were prepared separately in the ratio 1:1 in culture medium without penicillin and streptomycin and incubated for five minutes. Lipofectamine reagent was introduced in plasmid eppendorf and incubated for 20 minutes to promote the micelle formation. DNA plasmid was transfected into the cells cytoplasm for 6 hours and then was removed from culture medium, to avoid cellular toxicity, and cells were observed for the next three days.

RNA extraction and quantitative RT-PCR

Quantitative RT-PCR (qRT-PCR) was executed as described below. The total RNA was isolated from SH-SY5Y and SK-N-SH neuroblastoma cells using the RNeasy kit (Qiagen) and digested with the RNase-Free DNase set (Qiagen), according to the manufacturer's protocol. One microgram of total RNA was retrotranscribed into complementary DNA (cDNA) using murine leukemia virus reverse transcriptase (Promega Italia) and oligo(dT)15-18 as a primer (final volume: 12 µl). The oligonucleotide sequences of the primers used are as follows: TMOD1 forward primer 5'-TGTGAGGAAGAGGAGGCTTG-3', reverse primer 5'-AAAGGGTTTCATGCTGCCAG-3'; TMOD2 forward primer 5'-CTGGAGAAGGAGGCTTTGGA-3', reverse primer 5'-TACTCCAAGGACAGCTGCAA-3'; MAP2 forward primer 5'-CCCCTTGCTTCCCTGTAGAA-3', reverse primer 5'-ATTTCCTCCTGGCAACCTCA-3'; GAP43 forward primer 5'-TTCTTGGTGTTGT TATGGCAAG-3', reverse primer 5'-GAGGAAAG TGGACTCCCACAG-3'; TH forward primer 5'-ACACTTGTCCAGCTCTGACA-3', reverse primer 5'-CCGAGCTGTGAAGGTGTTTG-3'; DBH forward primer 5'-TTTCCCCTGSCCCTGAATCC-3', reverse primer 5'-TCTCTTTAGCTGCAGTGCCT-3'; SIN PHYS forward primer 5'-GGGCGTGACTTCAGACTCTC-3', reverse primer 5'-CAAGACCACCTTGGGTCCTA-3'; NSE forward primer 5'-GAGACAAACAGCGTT ACTTAG-3', reverse primer 5'-AGCTGCC CCTGCCTTAC-3'; CgA forward primer 5'-GCGG TGGAAGAGCCATCAT-3', reverse primer 5'-TC TGTGGCTTCACCACTTTTCTC-3'; GAPDH forward primer 5'- GAGTCAACGGATTTGGTCGT-3', reverse primer 5'-TTGATTTTGGAGGGATCTCG-3'. Amplification and detection were performed with the ViiA7™ real-time PCR System (Applied Biosystems); the fluorescence signal was generated by SYBR Green I. Samples were run in triplicate in a 12 µl reaction mix containing 6 µl 2x SYBR Green Master Mix (BioRad Italy), 12.5 pmol of each forward and reverse primer and 2 µl of diluted cDNA. The PCR program was initiated by 10 min at 95°C before 40 cycles, each for 15 s at 95°C and 1 min at 60°C. Gene expression levels were normalized to GAPDH expression and data are presented as the fold change in target gene expression in drug-treated cells normalized to the internal control gene (GAPDH) and relative to untreated cells. Results were estimated as Ct values; the Ct was calculated as the mean of the Ct for the target gene minus the mean of the Ct for the internal control gene. The Ct represented the mean difference between the Ct of untreated cells minus the Ct of treated cells. The *N*-fold differential expression in the target gene of drug-treated cells compared with untreated cells was expressed as $2^{-\Delta\Delta Ct}$. Data analysis and graphics were performed using Graph Pad Prism 6 software and the results of experiments run in triplicate.

Western blotting

Total cell lysates were prepared by scraping the cells in lysis buffer (50 mM Tris pH 7.6, 150 mM NaCl, 2 mM EDTA, 0.5% NP40 with a cocktail of protease and phosphatase inhibitors). For Western blotting analysis, 15 µg of total proteins were electrophoresed into 10% SDS-PAGE precast gel (Biorad Italy) and transferred to

nitrocellulose paper. Filters were incubated with anti-TMOD1 monoclonal primary antibody (Sigma-Aldrich, dilution 1:250), anti-TMOD2 polyclonal primary antibody (Santa Cruz Biotechnology, dilution 1:400) and anti-GAPDH primary antibody (Millipore, dilution 1:500) as loading control. After washing, membranes were incubated with IRDYe 680 RD goat anti mouse secondary antibody (LI-COR Biosciences, dilution 1:1500), IRDYe 680 RD goat anti rabbit secondary antibody (LI-COR Biosciences, dilution 1:1500), IRDYe 800 CW goat anti mouse secondary antibody (LI-COR Biosciences, dilution 1:1500) and IRDYe 800 CW goat anti rabbit secondary antibody (LI-COR Biosciences, dilution 1:1500) for 1 hour at room temperature and Odyssey® CLx Infrared Imaging System (LI-COR Biosciences) detected IR fluorescence. Densitometric analysis was performed by Odyssey quantification software.

Immunofluorescence analysis

Immunofluorescence analysis was executed as described below. SH-SY5Y and SK-N-SH cells were plated with a density of 75 X 10^3/well in a 24 wells plate, grown on glass coverslip (coated with poly-l-lysine, Sigma-Aldrich). Cells were fixed in ice-cold methanol (Sigma-Aldrich), then washed and incubated in Phosphate Buffered Saline (PBS, Sigma-Aldrich) containing 1% of Bovine Serum Albumin (BSA, Sigma-Aldrich) and 0.2% Triton X 100 overnight at 4°C with anti-TMOD1 monoclonal primary antibody (Sigma-Aldrich, dilution 1:100), anti-TMOD2 polyclonal primary antibody (Sigma-Aldrich, dilution 1:300), anti-βIII tubulin monoclonal primary antibody (Sigma-Aldrich, dilution 1:600) and anti-Ki-67 polyclonal primary antibody (Sigma-Aldrich, dilution 1:300). After rinses, cells were incubated with Alexa Fluor® 488 anti-mouse (Life Technologies, dilution 1:400), Alexa Fluor® 488 anti-rabbit secondary antibody (Life Technologies, dilution 1:400) and CY™3-conjugated anti-mouse secondary antibody (Jackson Immunoresearch Laboratories, INC, dilution 1:500) in PBS for 1 hour at room temperature. For morphological evaluation, slices were mounted and examined by a ZEISS LSM 510 META confocal laser-scanning microscope (Carl Zeiss, Germany). Images were processed using ZEN Microscope and Imaging Software (Carl Zeiss, Germany). The percentage of cells proliferation was determinate by counted the number of Ki-67 nuclei positive cells at least 10 fields for each treatment.

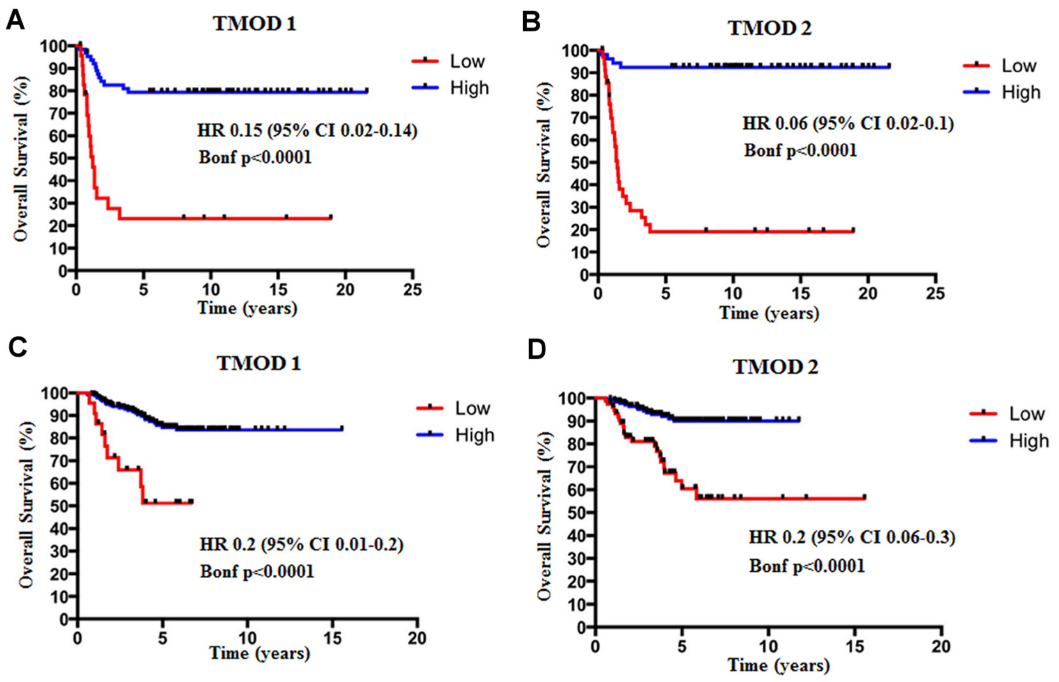

Supplementary Figure 1: Kaplan-Meier curves show overall survival in 88 **(A and B)** and 251 **(C and D)** patients' cohorts, respectively, cohort stratified by *TMOD1* (A and C) and *TMOD2* (B and D). Plots are entitled with the corresponding gene symbol. High expression (blue) and low expression (red) curves were compared by log-rank test and corrected for multiple hypotheses testing by Bonferroni method. Each plot reports the Bonferroni corrected p value (Bonf p), the hazard ratio and confidence interval of the stratification. A Bonf p smaller than 0.05 was considered a significant difference. HR: hazard ratio. CI: confidence interval.

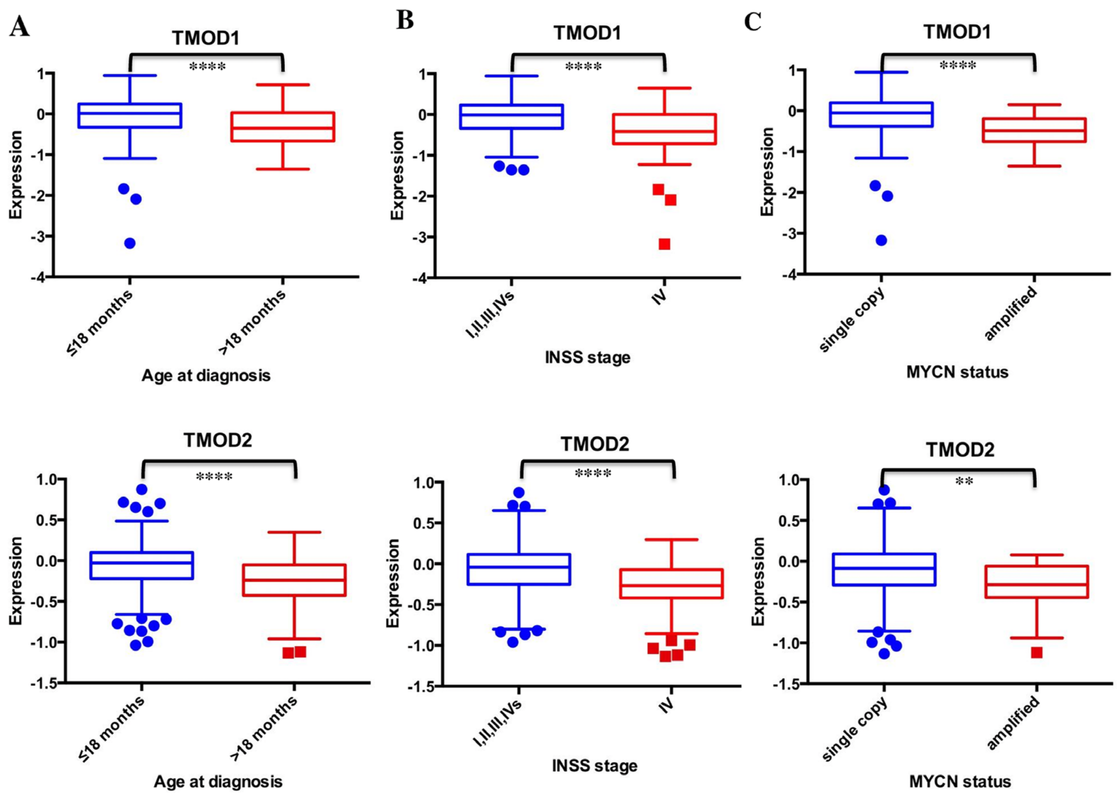

Supplementary Figure 2: Box and whisker plots of *TMOD1* and *TMOD2* in the populations defined by Age at diagnosis **(A)**, INSS stage **(B)** and *MYCN* status **(C)** risk factors. Data are relative to the patients in the 251 patients' cohort. Plots are entitled with the corresponding gene symbol. Significance of the difference between the means has been assessed by unpaired *t* test. A probability smaller than 0.05 was considered a significant difference. ** means $p<0.001$, *** means $p<0.0001$ and **** means $p<0.00001$. Age at diagnosis (≤ 18 months (blue) vs. > 18 months (red)), INSS stage (I, II, III, IVs (blue) vs. IV(red)) and *MYCN* status (single copy (blue) vs. amplified (red)) groups define the clinical and molecular sub-populations.

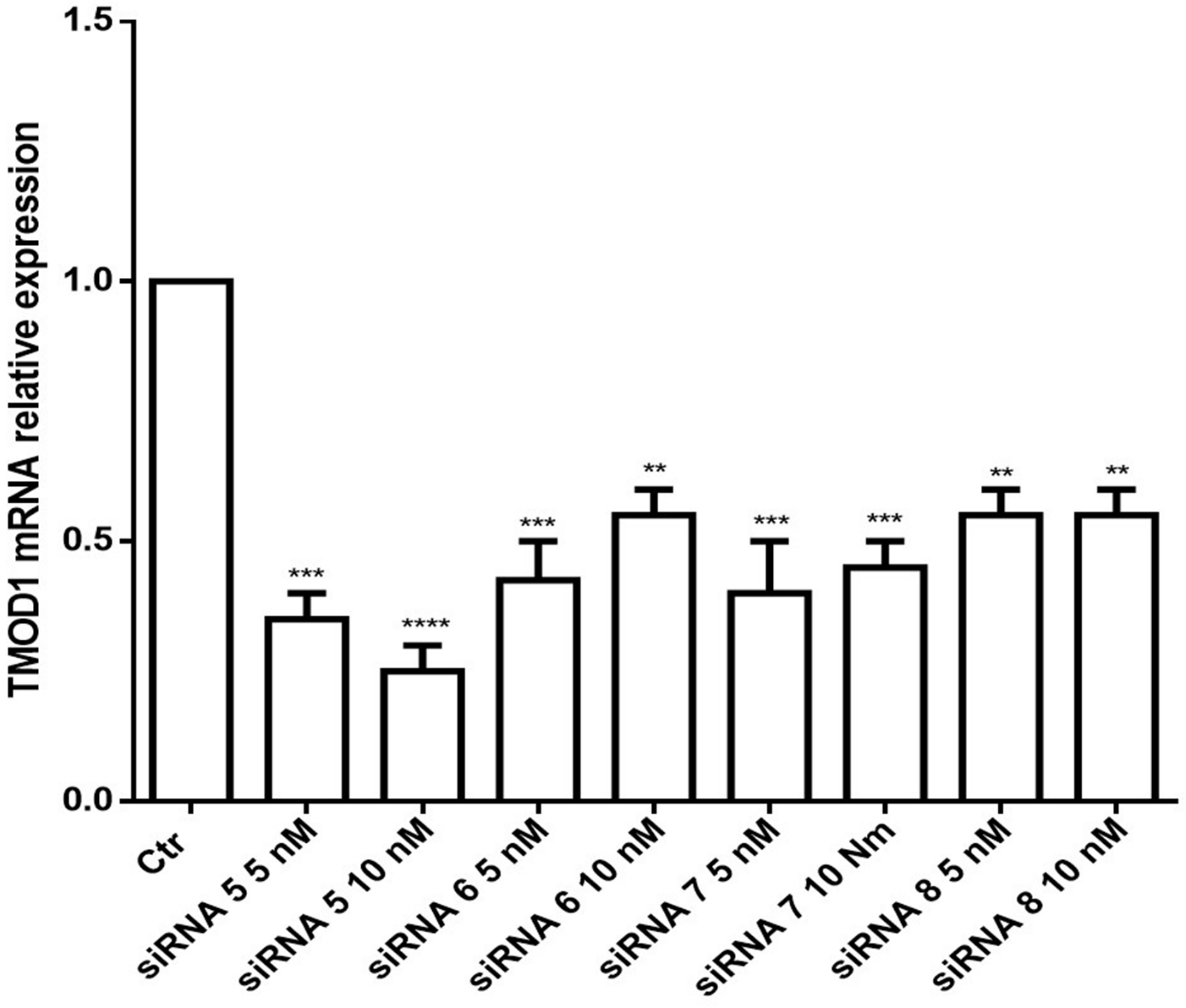

Supplementary Figure 3: qRT-PCR analysis of TMOD1 mRNA expression levels after different siRNA transient transfections. Neuroblastoma cell lines were transfected with four different siRNAs (siRNA 5, siRNA 6, siRNA 7, and siRNA 8) at two different concentration (5 nM, 10 nM) for 72 hours. The results obtained in the two cell lines are overlapping. **/***/**** means p< 0.0001 vs. control.

Supplementary Table 1: The table showed datasets utilized in the comparison analysis. [a] The data set name utilized for the comparison. Datasets are ordered in decreasing order of number of patients and they are relative to tumors tissues profiled by Affymetrix U133 plus 2 and normalized by MAS 5.0 algorithm. [b] Number of patients in the cohort. [c] Tumor type. [d] GEO (Gene Expression Omnibus) repository number for reproducibility.

See Supplementary File 1

Supplementary Table 2: Multivariate analysis of overall survival for *TMOD1* and *TMOD2*. Multivariate analysis was assessed by Cox regression in the 88 patients' cohort. Age at diagnosis, *MYCN* status and INSS stage risk factors have been discretized and values reported within parenthesis. HR=Hazard ration. CI=Confidence interval. INSS: international neuroblastoma staging system

See Supplementary File 2
